# Consensus document on the clinical application of invasive functional coronary angiography from the Japanese Association of Cardiovascular Intervention and Therapeutics

**DOI:** 10.1007/s12928-024-00988-5

**Published:** 2024-02-17

**Authors:** Taku Asano, Toru Tanigaki, Kazumasa Ikeda, Masafumi Ono, Hiroyoshi Yokoi, Yoshio Kobayashi, Ken Kozuma, Nobuhiro Tanaka, Yoshiaki Kawase, Hitoshi Matsuo

**Affiliations:** 1 Department of Cardiovascular Medicine, St. Luke’s International Hospital, 9-1 Akashi-cho, Chuo-ku, P.O. Box 104-8560, Tokyo, Japan; 2 Department of Cardiovascular Medicine, Gifu Heart Center, Gifu, Japan; 3 Department of Cardiology, Tokyo Medical University Hachioji Medical Center, Tokyo, Japan; 4 Department of Cardiovascular Medicine, Fukuoka Sanno Hospital, Fukuoka, Japan; 5 Department of Cardiovascular Medicine, Chiba University, Chiba, Japan; 6 Department of Cardiology, Teikyo University, Tokyo, Japan

**Keywords:** Functional coronary angiography, Angiography-derived FFR, Angiography-based FFR, Percutanous coronary intervention

## Abstract

Invasive functional coronary angiography (FCA), an angiography-derived physiological index of the functional significance of coronary obstruction, is a novel physiological assessment tool for coronary obstruction that does not require the utilization of a pressure wire. This technology enables operators to rapidly evaluate the functional relevance of coronary stenoses during and even after angiography while reducing the burden of cost and complication risks related to the pressure wire. FCA can be used for treatment decision-making for revascularization, strategy planning for percutaneous coronary intervention, and procedure optimization. Currently, various software-computing FCAs are available worldwide, with unique features in their computation algorithms and functions. With the emerging application of this novel technology in various clinical scenarios, the Japanese Association of Cardiovascular Intervention and Therapeutics task force was created to outline expert consensus on the clinical use of FCA. This consensus document advocates optimal clinical applications of FCA according to currently available evidence while summarizing the concept, history, limitations, and future perspectives of FCA along with globally available software.

**Graphical abstract:**

Overview and proposed clinical applications of functional coronary angiography (FCA). The FCA was developed according to computed fluid dynamics (CFD), considering the pressure drop across the coronary stenosis. CFD analysis was performed with a three-dimensional coronary model derived from angiography, allowing the calculation of the physiological index without the use of a pressure wire. Another direction of development in FCA is using artificial intelligence throughout the entire process, enabling “hands-free” FFR simulation. The advantage of the FCA is that it eliminates the use of a pressure wire, resulting in reduced invasiveness, shorter procedure times, and reduced medical costs. However, FCA requires high-quality angiography for a clear visualization of the lesion. In addition, the current version of FCA requires several manual corrections; thus, its reproducibility is limited. Further data on clinical outcomes after the use of FCA, such as percutaneous coronary intervention (PCI) with FCA guidance, are warranted. The consensus group of the Japanese Association of Cardiovascular Intervention and Therapeutics proposed the application of FCA in various clinical scenarios considering the advantages of this technology. QFR, quantitative flow ratio; iFR, instantaneous wave-free ratio; CCS, chronic coronary syndrome; ACS, acute coronary syndrome; MVD, multivessel disease; AMI, acute myocardial infarction.

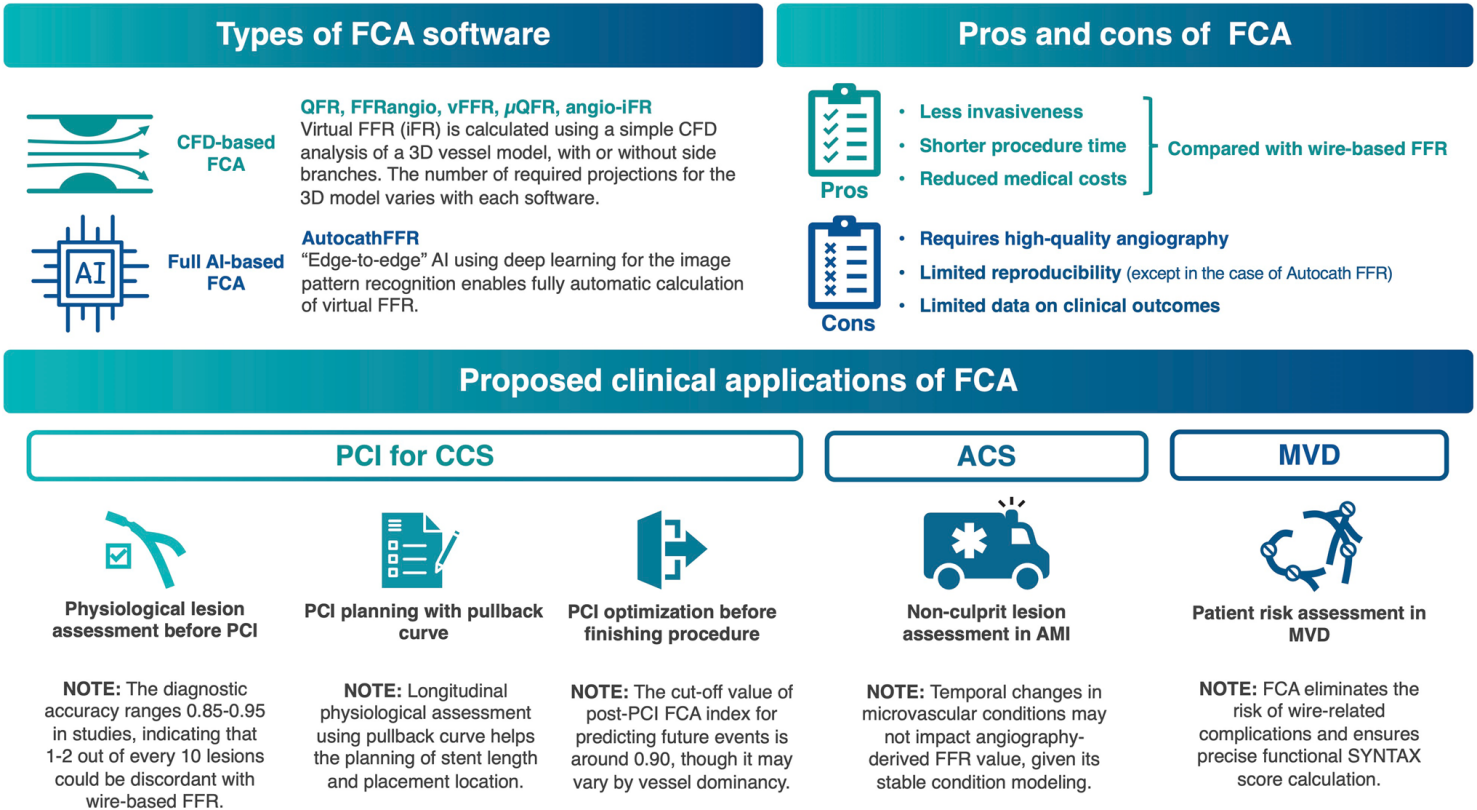

## Introduction

Physiological coronary artery assessment using a pressure wire enhances the efficacy of revascularization in reducing future adverse events in patients with coronary artery disease [1]. This approach ensures that only functionally significant lesions that potentially benefit from revascularization are treated appropriately, leading to improved outcomes and a decreased risk of revascularization-related complications. Previous studies have revealed the superiority of physiology-guided percutaneous coronary intervention (PCI) using fractional flow reserve (FFR) over angiography-guided PCI or medical therapy alone for composite adverse events, including urgent revascularization [2, 3]. According to these reports, FFR carries a Class 1A recommendation in the guidelines for determining the indication of PCI [4–6]. However, despite this, the global adaptation of FFR to guide decision-making for revascularization has remained low [1]. The issue is mainly ascribed to the cost of the pressure wire and adenosine, the time required to perform the FFR measurement, and the potential risk of complications related to the pressure wire [1].

Recently, invasive functional coronary angiography (FCA), an angiography-derived physiological index of the functional significance of coronary obstructions, has been employed worldwide for coronary artery disease in various clinical settings. FCA does not require a pressure wire or pharmacological hyperemia, thus potentially reducing the burden of cost, time, and complication risks associated with physiological assessments using invasive coronary angiography. With the growing evidence and clinical needs for FCA, this consensus document from the Japanese Association of Cardiovascular Intervention and Therapeutics reviews the currently available evidence on FCA and provides insights into its clinical application in various settings using globally available technologies.

## The “FCA” terminology

FCA has been called “angiography-derived FFR” or “angiography-based FFR” as this technology was developed to simulate an FFR value. However, herein, we refer to this technology as “FCA,” while avoiding the use of “FFR,” because we consider this as one of the angiographic parameters and a distinct parameter from FFR. FFR is defined as the ratio of the two flows (i.e., hyperemic coronary flow under stenotic conditions to that in the same coronary territory without stenosis) [7]. Conversely, FCA is derived from angiographic information, including the geometrical characteristics of the coronary arteries, such as the stenosis severity and length. The computation of the FCA involves mathematical models that approximate the relationship between blood flow and geometric conditions, necessitating several simplifications. These simplifications can lead to minimal discrepancies between the FCA and FFR values, although they are minimal. When the FCA is perceived as merely an FFR surrogate, these discrepancies may be misconstrued as errors in technology. However, when FCA is recognized as a novel distinct angiographic parameter with its own potential clinical significance, its value becomes apparent, similar to other modalities such as single-photon emission computed tomography and myocardial perfusion magnetic resonance imaging.

Conventional angiographic parameters represent the geometric characteristics of the coronary arteries at the maximal or minimal points. These parameters do not always represent the functional relevance of the coronary arteries [8]. However, just as the progressive burden of atherosclerotic plaques impedes coronary blood flow, the geometric characteristics of lesions are essential determinants of coronary function delivering blood to the myocardium. The development of this novel angiographic parameter aimed to better translate geometric lesion characteristics into coronary artery function. Accordingly, we combined “functional” with “coronary angiography” to name this technology, although the translation from geometry to function poses a great challenge. In a review article written by international experts in this field, they named this technology “FCA.” [9] However, this included the virtual FFR derived from coronary computed tomography angiography (FFR_CT_). Thus, we specifically refer to this technology derived from invasive coronary angiography as “invasive” FCA.

## The basic concept of FCA computation and its technological evolution

The concept of FCA calculation stems from the estimation of the pressure loss across the stenosis using computational flow dynamics (CFD) according to angiographic anatomical information. In the early 2010s, the initial iteration of the FCA was developed. Morris et al. reported the first successful development of a virtual FFR calculation workflow based on angiography in 2013 [10]. In this workflow, a virtual FFR was calculated by employing the mean aortic pressure for the upstream boundary condition and generic parameters for the downstream boundary condition, along with the Windkessel model. By conducting a time-varied three-dimensional (3D) CFD analysis, the Navier–Stokes equation was solved for the whole cardiac cycle based on a 3D vessel model reconstructed from rotational angiography. The virtual FFR values concurred substantially with the measured wire-based FFR values in their first-in-man study [10]. The workflow limitation is its lengthy calculation time. The CFD computation time was approximately 24 h per case, indicating that this workflow cannot be employed clinically in the catheter laboratory. However, in 2014, Papafaklis et al. introduced a fast and simple CFD-based virtual hemodynamic assessment model, known as the virtual Functional Assessment Index [11]. This innovative model solely relies on angiography and employs a simple quadratic equation derived from the Poiseuille and Bernoulli equations. It considers factors such as the pressure drop owing to viscous friction and flow separation. Remarkably, this model could provide a hemodynamic assessment in just 15 min.

**Fig. 1:**
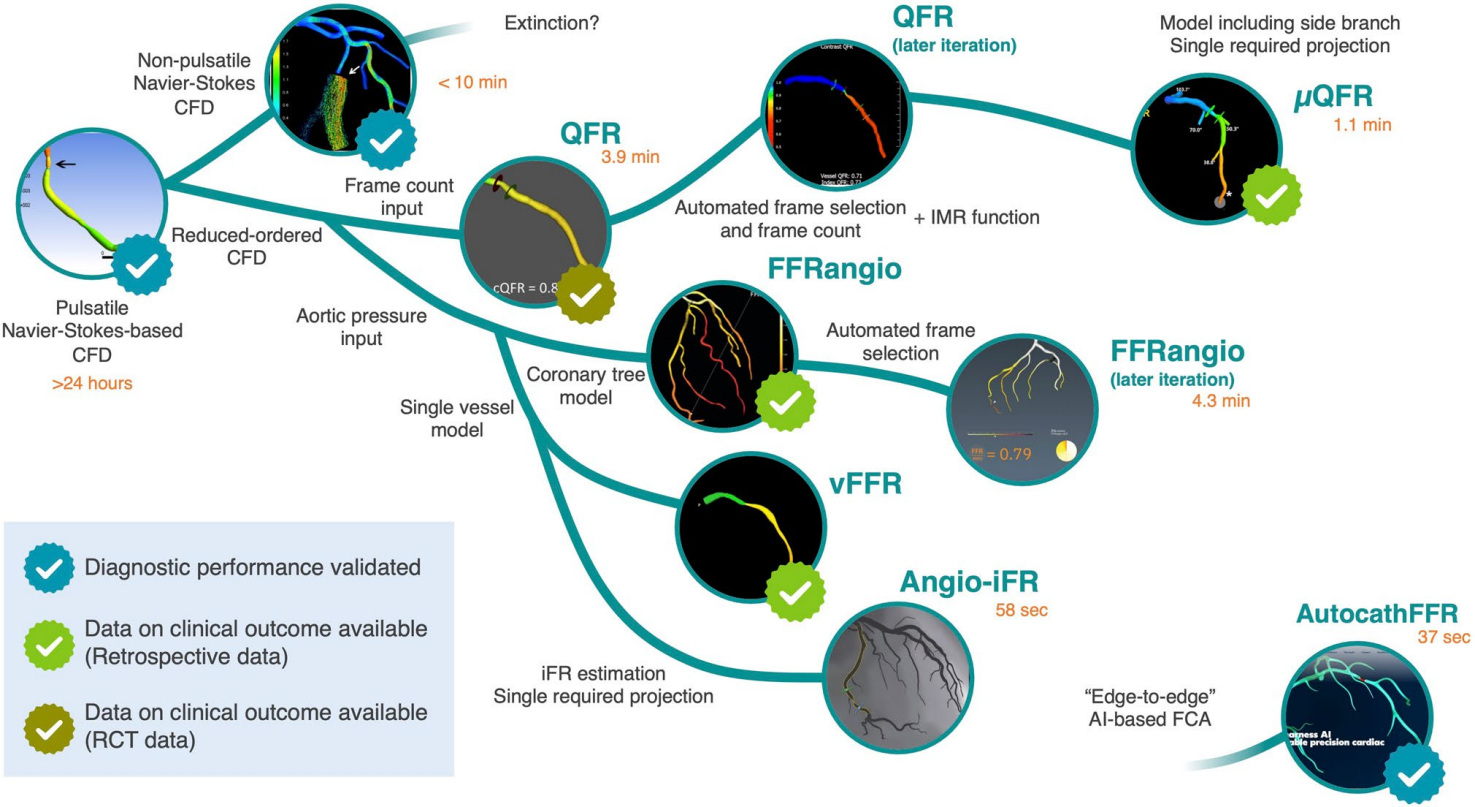
The evolution of FCA. FCA was first invented using sophisticated computed fluid dynamics (CFD) solving the Navier–Stokes equation based on three-dimensional angiography. This technology was developed significantly when reduced-order CFD analysis was employed. Quantitative flow ration (QFR) uses the frame-count method to determine the boundary conditions. The QFR was developed using automated procedures and an additional function to measure the index of microvascular resistance. The FFRangio, vFFR, and angio-iFR use aortic pressures to determine the boundary conditions. The FFRangio model included side branches for a more precise FFR simulation. µQFR is a novel software that requires a single projection for the vessel model, including side branches, with a significantly reduced procedure time (1.1 min). Another significant development is the advent of an “edge-to-edge” artificial intelligence-based FCA. This technology generates a virtual FFR using deep learning for image pattern recognition without CFD analysis

This groundbreaking simplification paved the way for FCA advancements (Fig. 1). In 2016, Tu and Kornowski furthered this development through the introduction of quantitative flow ratio (QFR) and FFRangio [12, 13]. Building upon the foundational work of Papafaklis, these approaches shared the fundamental concept of simplification. However, they offered unique perspectives on the inlet and outlet boundary conditions, thereby enhancing the practicality of the FCA. These technologies improved with the automation of the analytical procedure aimed at reducing the procedure time and improving the reproducibility of the technologies. As further evolutions of FCA, it has been elaborated with models including side branches, reducing the required projections and additional functions performing other physiological analyses, such as non-hyperemic pressure index (NHPI), index of microcirculatory resistance (IMR), and radial wall strain analysis [14–16]. The advent of the FCA implementing full-automatic analysis with “end-to-end” artificial intelligence (AI) is a recent significant evolution, enabling reproducibility improvement [17].

## Specific software of FCA

### Quantitative Flow Ratio® (QFR)

QFR^®^ (Medis Medical Imaging, Leiden, the Netherlands) is the first available software on the global market and has abundant evidence regarding diagnostic accuracy against wire-based FFR in numerous clinical settings and clinical outcomes after QFR-guided PCI in a large population (Table 1). The QFR calculation was based on the 3D quantitative coronary angiography (QCA) reconstructed from two angiographic projections with angles ≥ 25° apart and the volumetric flow rate calculated through contrast bolus frame count. 3D-QCA is conducted by defining anatomical markers (e.g., bifurcations) as reference points for two angiographic views for automated co-registration [18]. In the QFR software, the virtual FFR is computed based on pressure gradient accumulation within 6-mm-length vessel segments, estimated based on a simple tubular stenotic model where blood viscosity and flow separation were considered [12]. Using the frame count method, the rate of volumetric flow rate was evaluated [19].

**Table 1:**
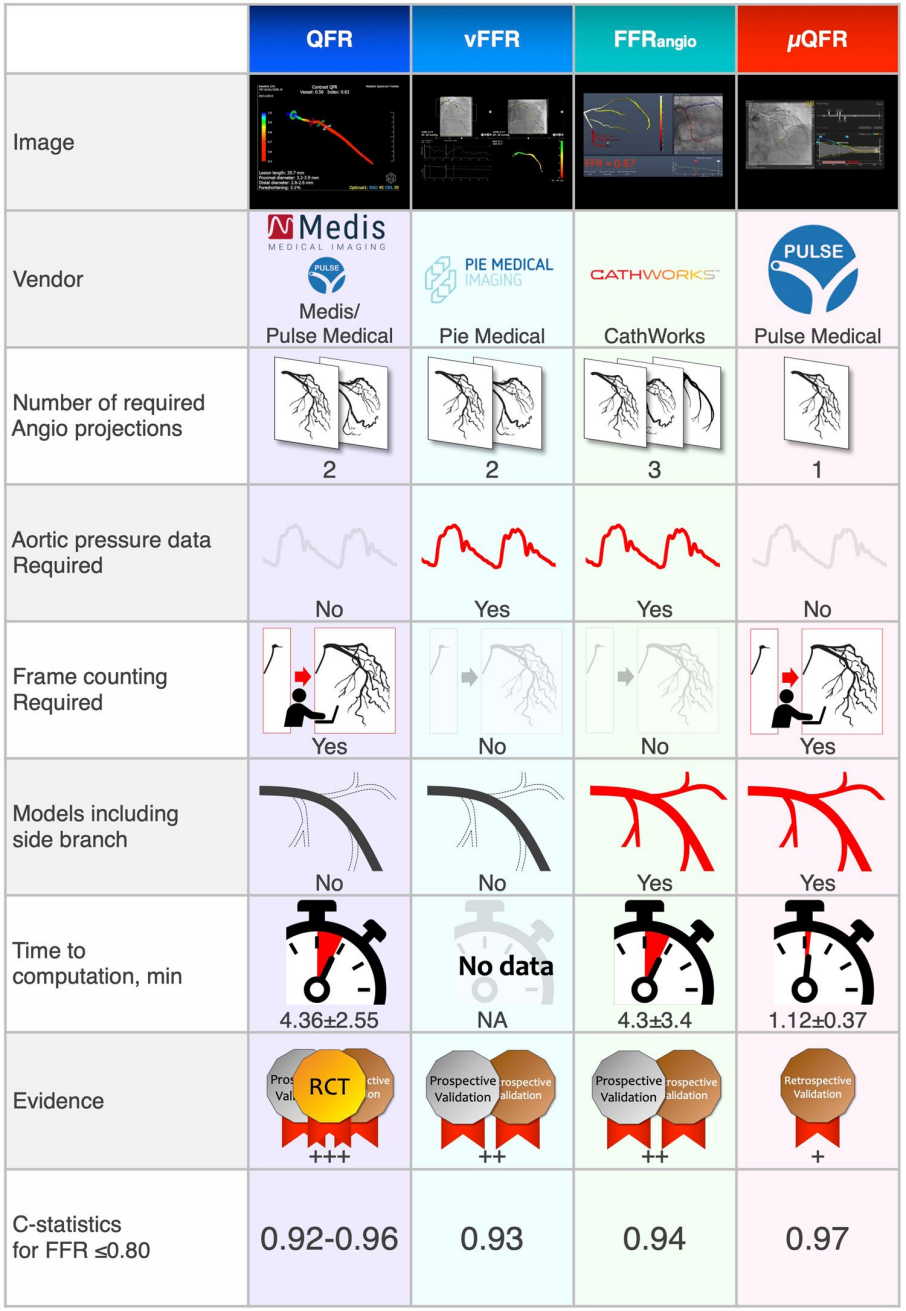
Comparative table of globally available FCA

*QFR* quantitative flow ratio, *FFR* fractional flow reserve

The software obtained CE Mark in 2017, followed by clearance from the Chinese National Medical Products Administration in 2018, the U.S. Food and Drug Administration (FDA) in 2019, and the Japanese Pharmaceuticals and Medical Devices Agency (PMDA) in 2023. The QFR analysis system is established by installing the software in a simple workstation (i.e., a conventional personal computer and an operating system) and therefore allows both online and offline analyses for clinical and research purposes. The latest version of the software (QAngio XA 3D 2.2) conducts QFR analysis using systematically automated procedures, including the detection of appropriate cine frames at the end-diastolic phase, vessel contour delineation, 3D angiography offset correction, definition of reference vessel segments, and execution of the frame count workflow. This software has been approved for clinical use in more than 40 countries, including the European Union.

The diagnostic performance of the QFR has been well examined in a considerable number of studies using FFR as a reference. The FAVOR II China study explored the diagnostic performance of online QFR using FFR as a reference for 332 vessels with intermediate stenoses [20]. In the study, the correlation coefficient between QFR and FFR was reported as 0.86 (*p* < 0.001), while the mean difference was − 0.01 (standard deviation = 0.06). The accuracy, sensitivity, specificity, positive predictive value, and negative predictive values of QFR for an FFR of ≤ 0.80 were 92.7%, 94.6%, 91.7%, 85.5%, and 97.1%, respectively. The area under the receiver operating characteristic curve (AUC) was 0.96. A patient-level meta-analysis of 819 patients with 969 vessels from major studies (i.e., FAVOR Pilot, WIFI II, FAVOR II China, and FAVOR II Europe-Japan) reported the pooled sensitivity, specificity, positive predictive value, and negative predictive value of 84%, 88%, 80%, and 95%, respectively. The pooled AUC was 0.92 [21].

### FFRangio™

FFRangio™ (CathWorks, Kfar-Saba, Israel) is a unique technology that provides 3D functional angiography mapping of the entire coronary artery (Table 1). Using routine coronary angiograms, FFRangio is based on 3D reconstruction of the entire coronary tree. This reconstruction is followed by a computer-based rapid flow analysis, which allows the calculation of FFRangio within a few minutes through automated processing. To achieve a 3D reconstruction of the entire coronary tree, at least three different angiographic projections are required. This system was granted FDA market clearance in 2018. Subsequently, it also obtained the CE mark in Europe, Medical Devices and Accessories approval in Israel, and PMDA approval in Japan.

3D reconstruction of the coronary tree is based on at least three known angiographic projections, and the process involves epipolar ray tracing and topology-preserving constraints. This is achieved automatically by reconstructing the geometry of the tree, including the centerlines and cross-sections at each point, while preserving the exact topology. The resulting coronary tree can emerge using a triangular mesh and rendered to create a 3D coronary model. The system scans the entire reconstructed coronary tree in 3D and analyzes each branch and bifurcation (or trifurcation) to identify narrow regions (stenoses) after 3D reconstruction. A hemodynamic evaluation was then conducted, considering the contribution of each narrowing to total resistance to flow. Subsequently, a lumped model was built, and the impact of certain vessels on the overall resistance influenced their contribution to flow control. The resistance of each vessel was estimated using Poiseuille’s law, according to its length and diameter. The accumulated volume of the coronary vessels and the total coronary length, calculated from the 3D reconstruction, allowed an estimation of the normal supply derived from the microcirculatory bed resistance. The lumped model solution based on the inlet and outlet boundary conditions allowed for the evaluation of the flow rate ratios for stenosed versus healthy coronary trees. A color-mapped mesh was generated to display the FFR values at each location in the coronary tree [13].

A pooled analysis of five prospective cohort studies on FFRangio involving 700 lesions in 588 patients was conducted [22]. FFRangio demonstrated an excellent diagnostic performance across various subgroups of patients and lesions. The diagnostic accuracy, sensitivity, specificity, positive predictive value, and negative predictive value were 93%, 91%, 94%, 91%, and 94%, respectively. A strong correlation was found between FFR and FFRangio with a coefficient of 0.83 (*p* < 0.001) and a minimum difference between FFR and FFRangio (0.00 ± 0.058). FFRangio also showed high diagnostic performance across various patient characteristics (age, sex, clinical presentation, body mass index, and diabetes) and lesion characteristics, including calcification, tortuosity, and lesion location [23]. The time required to measure FFRangio and FFR was evaluated for multivessel disease [24]. It took significantly less time for FFRangio (9.6 ± 3.4 min per patient and 4.3 ± 3.4 min per lesion) than that for FFR (15.9 ± 8.9 min per patient and 6.9 ± 5.6 min per lesion) (*p* < 0.001).

### CAAS vFFR

The CAAS vessel FFR (vFFR; Pie Medical Imaging BV, Maastricht, the Netherlands) is a globally available FCA (Table 1). A coronary artery is 3D reconstructed by exporting two orthogonal angiography images (with angles ≥ 30° apart) to the CAAS workstation. After the 3D reconstruction, the vFFR value is calculated using the invasively measured aortic root pressure as the inlet for the boundary condition. In addition to functional lesion information, this workstation provides anatomical lesion information (percentage diameter stenosis, minimal lumen diameter, reference lumen diameter, minimal lumen area, and lesion length).

The diagnostic performance of the vFFR in various scenarios was thoroughly examined. The Fast Assessment of STenosis severity (FAST) study retrospectively evaluated 100 lesions from 100 patients and evaluated the diagnostic accuracy of vFFR to predict FFR ≤ 0.80 [25]. The diagnostic accuracy was 93%, and a good linear correlation between FFR and vFFR was reported (*r* = 0.89; *p* < 0.01), with a low inter-observer variability (*r* = 0.95, *p* < 0.001). Based on the results of the FAST study, CAAS vFFR received FDA market clearance and obtained CE mark and PMDA approval. The FASTII study is an international multicenter trial that prospectively validates the diagnostic accuracy of vFFR in both on-site and core laboratory settings [26]. In this study, 334 patients from six countries (the Netherlands, Germany, Italy, France, the United States, and Japan) were enrolled. The diagnostic accuracy to predict an FFR ≤ 0.80 for both on-site and core laboratory setting was very high (91% and 93%, respectively). A retrospective study reported that vFFR provided high discrimination for coronary stenosis (area under the curve 0.80, 95% CI 0.70–0.90), which was comparable to that of non-hyperemic pressure ratios [27].

## General remarks on the clinical application of FCA

**Fig. 2:**
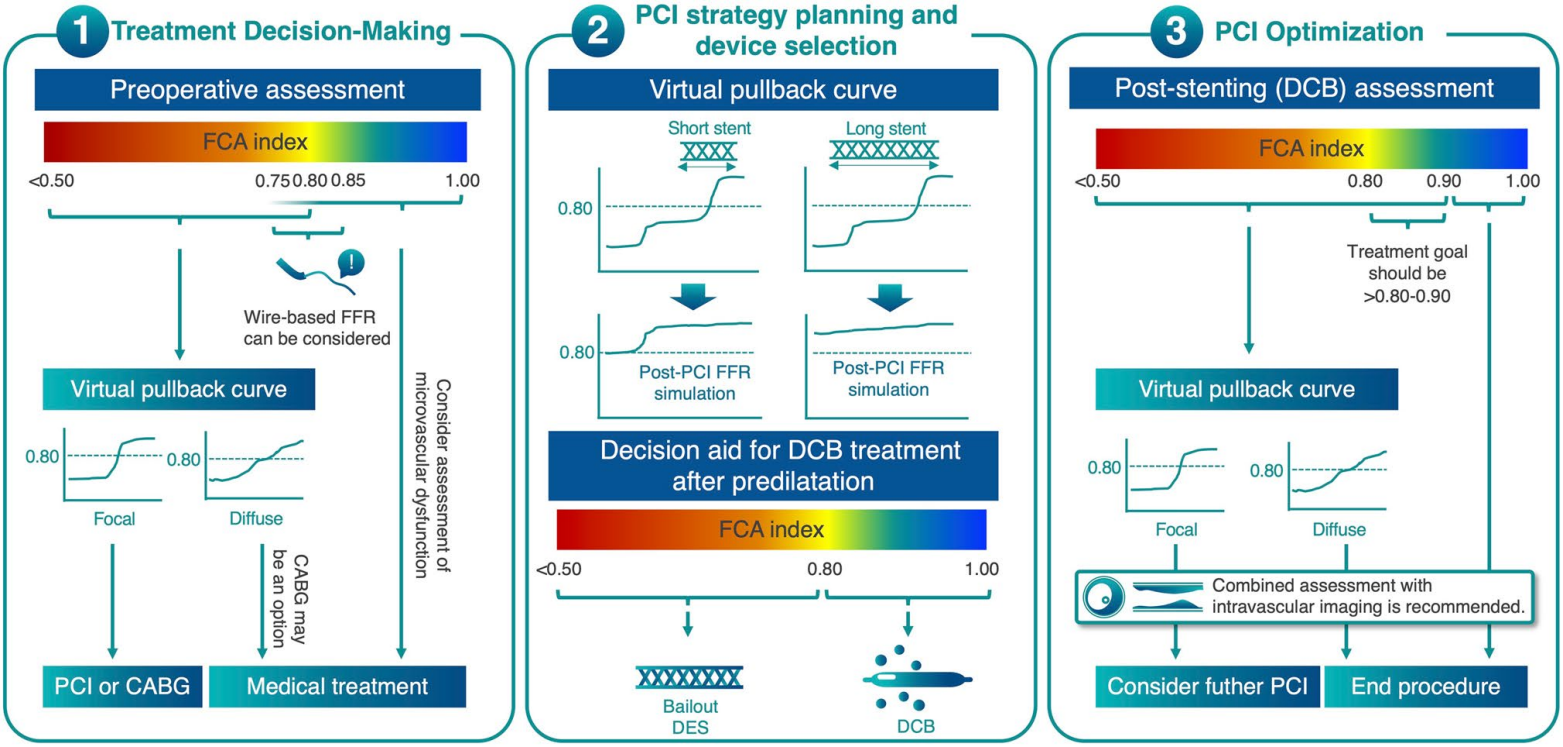
The proposed algorithm of peri-PCI physiological assessment based on FCA. *FFR* fractional flow reserve, *PCI* percutaneous coronary intervention, *CABG* coronary artery bypass graft, *DES* drug-eluting stent, *DCB* drug-coated balloon

Based on compiled evidence showing substantial diagnostic accuracy in validation studies, FCA has exhibited promise for clinical use in various clinical settings. Several studies have reported their utility and applicability in different clinical scenarios. These studies explored the use of FCA in diverse patient populations including those with chronic and acute coronary syndromes (ACS), multivessel disease, and severe valvular disease. One fundamental conceptual advantage of FCA is that it enables operators to conduct vessel-level physiological assessments without the need for a pressure wire, simultaneously with invasive coronary angiography in the catheter laboratory. This advantage of the FCA can significantly improve the accessibility of functional assessments. Improved accessibility can be highlighted at different stages of PCI (Fig. 2) as follows:

1. *Treatment decision-making before PCI:* FCA enables assessment of the hemodynamic significance of lesions prior to PCI without the need for a pressure wire. This physiological information aids in treatment decision-making, such as determining the need for revascularization or optimal medical treatment strategies for individual lesions or vessels. This decision-making process involves longitudinal physiological assessments of a vessel with virtual pullback to estimate PCI efficacy.
2. *PCI strategy planning and device selection:* Virtual pullback aids in building revascularization strategies such as short or long stenting if revascularization is indicated. FCA can be used to plan and modify treatment strategies in real time. By providing immediate feedback on the functional significance of lesions, operators can make informed decisions regarding lesion preparation, device selection, and optimization techniques without a pressure wire. The wire-based FFR assessment is proposed for the decision-making of the “stent-less” strategy using a drug-coated balloon (DCB), where an FFR > 0.80 after the pre-dilatation is an indication of DCB-only treatment in de novo lesions [28, 29]. Theoretically, in this clinical context, FCA can be also employed. However, it should be acknowledged that the efficacy of FCA on DCB treatment has not been evaluated.
3. *PCI optimization and prognostic assessment after the procedure:* FCA can be used for PCI optimization after stent implantation and employed after PCI to assess the prognostic significance of the treated vessel. Through the evaluation of residual functional impairment and considering the overall vessel-level physiology, clinicians can gain insight into the long-term prognosis and potentially guide further management or follow-up strategies.

By offering accessible and timely functional assessments at different stages of PCI, FCA contributes to enhanced treatment planning, improved procedural outcomes, and better prognostic evaluation in patients undergoing coronary interventions.

## Clinical applications of FCA in specific clinical scenarios

### FCA as a decision aid for revascularization

FFR is widely utilized to determine indications for revascularization of de novo intermediate lesions in patients with chronic coronary syndrome in routine clinical practice. As described above, the FCA was validated using the FFR as a reference standard for these lesions. In validation studies, FCA accurately discriminated lesions that were potentially from revascularization leading to improved clinical outcomes, while the AUC values of FCA for predicting an FFR of ≤ 0.80 consistently revealed a substantial level of performance, approximately around 0.90 [21, 22, 30]. Currently, there is paucity of clinical outcomes after PCI with FCA guidance, and therefore, these are of paramount interest. A prospective observational study examining clinical outcomes after FFRangio-guided PCI in 492 patients (53.4% were in an ACS setting) reported that the 1-year incidence of major cardiac adverse events (MACE), a composite of cardiovascular mortality, myocardial infarction (MI), and repeat revascularization, was 4.1% in patients undergoing PCI, while the MACE incidence of patients in whom PCI was deferred according to the FFRangio value was 2.5% [23]. The authors concluded that the incidence after FFRangio-guided PCI was substantially low in reference to an MACE rate of 6.3%, which was noted in a pooled analysis of five large international registries [31]. The FAVOR III China randomized controlled trial (RCT) compared the incidence of clinical outcomes between QFR- and angiography-guided PCI in 3,847 patients with coronary artery disease [32]. In that trial, 1-year MACE occurred in 5.8% of patients in the QFR-guided group and in 8.8% of patients in the angiography-guided group (hazard ratio [HR]: 0.65 [95% confidence interval {CI}: 0.51–0.83], *p* = 0.0004). This difference was driven by fewer MIs and ischemia-driven revascularizations in the QFR-guided group than those in the angiography-guided group. An ongoing large-scale randomized controlled trial (FAVOR III Europe–Japan trial) will investigate the non-inferiority of QFR-guided PCI to FFR-guided PCI in terms of clinical outcomes in 2000 patients with coronary stenoses [33].

### The longitudinal functional assessment of coronary artery disease using virtual pullback curve of FCA

In addition to the functional significance of the lesion, the longitudinal extent of functional disease plays a crucial role in determining the indication for revascularization. The pullback curve of a physiological index, such as the FFR and instantaneous wave-free ratio (iFR), indicates the longitudinal gradient pattern of the physiological impact in a vessel. Several studies have reported that a gradual reduction in pressure before PCI is linked to a smaller improvement in the physiological index following stent implantation than an abrupt decline [34, 35]. Collet et al. introduced a metric called the pullback pressure gradient (PPG) index, which considers both the magnitude of pressure losses and the extent of functional disease based on the FFR pullback curve [36]. This index quantifies the longitudinal distribution of pressure loss and effectively distinguishes between focal and diffuse coronary artery diseases. Similarly, the FCA software provides a virtual pullback curve to facilitate the longitudinal functional assessment of disease distribution. Dai et al. reported the feasibility of the PPG index calculated from the QFR virtual pullback curves [37]. In a post-hoc analysis of the PANDA III trial, it was found that in vessels with a high QFR-derived PPG index (indicating focal disease), PCI led to improved outcomes in terms of vessel-oriented adverse events. However, for vessels with a low PPG index (indicating diffuse disease), PCI did not provide the same benefit and demonstrated a comparable risk of adverse events as vessels were managed conservatively [37].

Longitudinal physiological information along a vessel can also be used for PCI planning and optimization. Kikuta et al. reported on the accuracy of the predicted post-PCI iFR derived from the iFR pullback curve in the iFR GRADIENT registry [38]. Compared with angiography alone, iFR pullback altered the number and length of treated lesions in approximately 31% of patients. Several FCA software programs simulate the residual FFR after PCI as computed from pre-PCI coronary angiograms. Studies have postulated the potential of the virtual residual FFR function to accurately predict the real post-PCI FFR [39–42].

### Hybrid approach combining FCA and wire-based FFR

In validation studies of FCAs, mismatches in functional significance, determined by a cutoff of 0.80, were noted between FCA and wire-based FFR [21, 25, 26, 43]. These mismatches were found to occur in the intermediate zone, specifically between 0.75 and 0.85. Several retrospective studies indicated that a hybrid approach, in which wire-based FFR was used only when FCA fell in the intermediate zone (e.g., 0.77–0.87), improved the diagnostic performance and reduced the need for more than half of the pressure wires [44–46]. The approach potentially enhances physiological assessment accuracy using FCA while reducing the need for pressure wires. This reduction not only alleviates the potential burden of complications, but also helps mitigate the high costs associated with revascularization procedures. However, the efficacy of this hybrid approach is yet to be validated through prospective studies, and there are unresolved concerns regarding medical insurance coverage for the simultaneous use of both physiological assessments in certain countries.

### Post-PCI FCA as a predictor of long-term clinical outcomes

Wire-based FFR immediately after stenting (post-PCI FFR) has been used for physiological assessment of residual stenoses and has been reported to be associated with long-term outcomes [47–49]. In line with wire-based FFR, post-PCI FCA is also expected to have a certain impact on long-term outcomes. A notable advantage of post-PCI FCA is that it eliminates the need to exchange a guidewire with a pressure wire, thereby simplifying the post-PCI functional assessment process. Kogame et al. studied the impact of post-PCI QFR on the 2-year vessel-oriented composite end point (VOCE), a composite of vessel-related cardiac death, vessel-related MI, and target vessel revascularization, in the post-hoc analysis of the SYNTAX II trial [50]. In the trial, a lower post-PCI QFR (< 0.91) was significantly associated with a higher incidence of 2-year VOCE (HR: 3.37, [95% CI: 1.91–5.97], *p* < 0.001). Similarly, the HAWKEYE prospective study elucidated that a post-PCI QFR ≤ 0.89 was associated with an increased risk of 2-year VOCE (HR: 2.91 [95% CI: 1.63–5.19], *p* < 0.001) [51]. As for other FCA software, a retrospective cohort study (FAST OUTCOME) examined the association between post-PCI vFFR and TVF at 5 years. In that study, vessels in the lower (vFFR < 0.88) and middle tercile (vFFR 0.88–0.93) had a higher risk of TVF as compared to vessels in the upper tercile (HR: 1.84 [95% CI: 1.15–2.95], *p* = 0.011, and 1.58 [95% CI: 1.02–2.45], *p* = 0.040, respectively) [52].

Although further research is underway to establish robust evidence for post-PCI FCA, its potential to simplify physiological assessments after stenting can be widely advocated for enhancing patient safety and cost-effectiveness.

### Physiological assessment of non-culprit lesions in patients with acute MI using FCA

In daily clinical practice, the decision regarding additional revascularization of non-infarct-related arterial lesions in patients with ST-segment elevation MI (STEMI) often poses a significant challenge. One of the important indicators for decision-making is vessel-level physiological data of non-infarct-related artery lesions. Previous studies have provided evidence supporting the superiority of FFR-guided complete revascularization over culprit-only revascularization in terms of MACE incidence [53, 54]. FCA has the potential to eliminate the requirement for exchanging guide catheters for the physiological assessment of the opposite coronary system, including the procedure of switching from a guide wire to a pressure wire. The diagnostic performance and feasibility of FCA for non-infarct-related arterial lesions have been examined in several studies. Lauri et al. reported that QFR has good diagnostic accuracy in evaluating the functional significance of non-infarct-related artery lesions during primary PCI, similar to the accuracy observed in stable patients (AUC 0.91 vs. 0.94, *p* = 0.50) [55]. A retrospective analysis involving 617 patients with STEMI demonstrated that the rate of the 5-year composite end point, including cardiac death, spontaneous non-target vessel MI, and clinically indicated non-target vessel revascularization, was significantly higher in patients with QFR ≤ 0.80 as compared to those with QFR > 0.80 (HR: 7.33 [95% CI: 4.54–11.83], *p* < 0.001) [56]. In a retrospective study including 441 patients with STEMI with multivessel disease, the discordance between vFFR-oriented functional judgment and the actual treatment strategy (PCI or deferral) was correlated with increased vessel-related adverse events [57].

It is noteworthy that in the comparison between FFR-guided and angiography-guided complete revascularization, the benefit of FFR-guided complete revascularization has been controversial in patients with acute MI. The FLOWER-MI RCT revealed that an FFR-guided strategy did not have a significant benefit over an angiography-guided strategy in terms of the risk of death, MI, or urgent revascularization at 1 year in patients with STEMI who underwent complete revascularization [58]. Conversely, the FRAME-AMI RCT revealed that FFR-guided complete revascularization was superior to angiography-guided complete revascularization in reducing the risk of death, MI, and repeat revascularization in patients with acute MI [59]. Regarding the results of a recent retrospective study, a plausible hypothesis was suggested to highlight the rationale for the failure of the FLOWER MI trial to demonstrate the efficacy of FFR in patients with STEMI. In addition, this study highlights the potential advantages of FCA in an acute setting. The study was conducted as a post-hoc analysis of the REDUCE-MVI study, in which the FFR value of non-infarct-related artery lesions measured 30 days after primary PCI significantly decreased as compared to FFR measured immediately after the primary PCI (0.85 at follow-up vs. 0.88 at baseline, *p* = 0.001) [60]. However, this time-dependent mismatch was not noted in QFR, remaining relatively constant (0.83 at follow-up vs 0.84 at baseline, *p* = 0.310). The FFR has been reported to potentially underscore the true functional impact of non-infarct-related artery stenosis when measured in ACS [31]. This could be explained by the failure to achieve maximum hyperemia due to the transient impairment of the microcirculation secondary to increased microvascular vasoconstriction, blunted hyperemic response, and elevated left ventricular filling pressure [61–63]. Nevertheless, QFR (including other software) was developed with the boundary condition modeled based on stable coronary disease, in which the hyperemic flow estimation was calculated based on generic numbers generated from the stable condition [12]. Thus, it can be hypothesized that the FCA is less susceptible to the transient impairment of microcirculation in the myocardium in ACS. This hypothesis should be addressed in future studies.

### Risk assessment in patients with multivessel disease using FCA

Vessel-level physiological information has the potential to improve the prognostic capability of the risk score based on anatomical information in patients with multivessel disease (MVD), which is represented by the SYNTAX score. The SYNTAX scoring system is an anatomical scoring system based on coronary angiography that is associated with long-term outcomes after PCI in patients with MVD or left main disease [64]. Nam et al. reported that the functional SYNTAX score, which was the recalculation of the SYNTAX score by only counting ischemia-producing lesions detected by an FFR of ≤ 0.80, yielded a better discriminative ability for the risk of 1-year MACE in patients with MVD as compared to anatomical SYNTAX score [65]. However, a challenge arises with the functional SYNTAX score when measuring the FFRs of multiple vessels as operators must consider the risk of complications related to the pressure wire and prolonged procedure time.

FCA has the potential to eliminate this issue. In patients with MVD (average anatomical SYNTAX score 13 ± 3.9), Omori et al. reported that the functional SYNTAX score derived from FFRangio reduced the point of the score by excluding non-ischemia-producing lesions being comparable to the functional SYNTAX score derived from wire-based FFR (functional SYNTAX score: 5.3 ± 6.8 vs. 5.6 ± 6.8) [24]. A substantial correlation existed between the two functional SYNTAX scores. In that study, the procedure time for measuring FFRangio per patient was significantly shorter than that of wire-based FFR (9.6 ± 3.4 vs. 15.9 ± 8.9 min). In the post-hoc analysis of the SYNTAX II trial, the functional SYNTAX score, derived from the QFR, showed improved prognostic capability for the 2-year patient-oriented composite end point (POCE) in patients with MVD compared to the anatomical SYNTAX score. The functional SYNTAX score derived from the QFR showed a significant net reclassification improvement of 0.32 (*p* < 0.001) and had a higher AUC than the anatomical SYNTAX score for predicting the 2-year POCE (0.68 vs. 0.56, *p* = 0.002) [66]. Another post-hoc study also reported improved discrimination ability of the functional SYNTAX score derived from QFR compared to the anatomical SYNTAX score in patients with MVD and left main disease [67].

FCA may enhance the practicality of physiology-based risk assessment in patients with MVD to determine the optimal treatment strategy, including the mode of revascularization (PCI or coronary bypass graft). However, the efficacy of physiologically guided treatment decision-making on clinical outcomes has not been fully evaluated in patients with MVD. Further investigations, including studies evaluating the efficacy of FCA-based treatment decision-making on long-term outcomes in patients with MVD, are warranted.

### The potential usage of FCA in other clinical scenarios

The FCA can potentially be utilized without the use of a pressure wire in other clinical scenarios, such as severely tortuous vessels, in-stent restenosis, and severe aortic stenosis [68–70]. However, owing to the limited number of reports, these methods require further evaluation.

## Practical considerations for FCA analysis

In clinical practice, a precise analysis is of paramount importance for the appropriate use of FCA. Although each software is being developed to elaborate its accuracy and reproducibility while balancing its practicality, users should acknowledge several considerations for FCA analysis. An important factor in determining the precision of FCA is coronary angiography quality [71]. For every type of software, operators should prospectively pay attention to acquiring high-quality coronary angiography which requires an interested segment of the vessel filled by contrast medium without overlapping or foreshortening in multiple projections obtained with angles of ≥ 25°–30° apart. To ensure an accurate co-registration in 3D vessel reconstruction, operators must carefully monitor and adjust heart displacement caused by respiratory movements. For a qualified coronary angiography, recording cine angiography with a frame rate of ≥ 15 frame/s using ≥ 5 Fr diagnostic catheters following nitroglycerin injection is recommended. The following section presents specific considerations for each software package.

### QFR

To ensure reproducibility of the analysis, the analytical procedures of QFR were systematically automated in the latest version of the software (version 2.2). Thus, operators require minimal manual corrections when considerable errors are observed. However, several crucial factors that significantly affect QFR calculation results are worthy of attention. These factors include the selection of appropriate angiographic frames in the end-diastolic phase (definition of end-diastolic frames), accurate vessel lumen definition (delineation of lumen contours), precise reference vessel modeling through interpolation using the proximal and distal segments of the target vessel (definition of reference diameters), and determination of volumetric flow (frame counting). Moreover, it is vital to highlight that when analyzing major bifurcations, special consideration is warranted because QFR calculation is based on a single tubular model. The diagnostic performance of QFR was impaired in bifurcated lesions [66]. The diameter of the vessels abruptly decreased because of the distribution of the required blood flow at the branching points of the blood vessels [72]. In this specific anatomical condition, estimating the pressure drop and volumetric flow in the model generated by the QFR software can be challenging. If there is a significant difference in the diameter between the mother and daughter vessels, defining the analytical segment without including major bifurcations (e.g., left main coronary artery) is recommended for the operators. However, this exception can occur if there are no intermediate lesions upstream of the bifurcation.

### FFRangio

Coronary angiography was conducted according to standards of care. The cine frame rate for the angiograms was set at a minimum of 10 f/s. A high resolution is required for the images (≥ 700 × 700) without being highly compressed. Diagnostic angiograms are obtained using different projections with angles ≥ 30° apart, finally depending on the operator’s discretion. To ensure an accurate visualization of the coronary tree, the entire vessel was more carefully imaged than other FCA, with adequate contrast opacification, avoiding vessel overlap, and without panning the table or moving the image intensifier during the procedure. These precautions should be exercised to obtain high-quality angiographic images suitable for analysis and 3D reconstruction required for FFRangio calculations.

Correcting the vessel contours in the analysis of FFRangio may be warranted, similar to other FCA methods, although this task is reduced with high-quality images. Unlike other FCAs, FFRangio analyzes the entire coronary artery and requires the analyst to specify the side branches of interest. Although the system automatically identifies the major side branches, the analyst must manually designate the remaining side branches. Insufficient identification of side branches in each image can lead to incomplete 3D reconstruction of the branches, potentially affecting the accuracy of the analysis results.

### CAAS vFFR

The vFFR analysis consists of two steps: (1) proper image acquisition and (2) 3D analysis. Operators must consider these factors when acquiring coronary angiographic images. The two angiograms used for 3D construction should be at least 30° apart (90° is recommended). 3D construction requires the extraction of precise vessel contours, selection of the time phase, and adjustment of the common image point (CIP), a landmark indicating the same anatomic location. Contour detection is performed almost automatically, and manual correction may be required if the contour detection is not optimal. Temporal alignment of the two images was automatically attained by ECG triggering to synchronize them in the same phase of the cardiac cycle. The software automatically determines the CIP, whereas manual correction is required when discrepancies occur. The center of the stenosis helps determine the CIP location.

## Limitations of FCA

As described above, the use of FCA has been evaluated in several studies conducted under various clinical scenarios. However, most of these studies are retrospective. Further prospective studies to evaluate the efficacy and safety of FCA on clinical outcomes should confirm the validity of the concept of the “wire-less” physiological assessment in the context of coronary revascularization.

Reportedly, the current version of the FCA may have limitations under specific conditions. Mejía-Rentería et al. evaluated the influence of microvascular dysfunction measured with wire-based IMR on the diagnostic performance of QFR, with FFR as a reference standard. In this study, a significantly lower classification agreement and AUC of the QFR were found in the high-IMR group than that in the low-IMR group [73]. Other scenarios, such as the use of FCA in the left main coronary artery, aorto-ostial lesions, and severely tortuous vessels, have not been fully evaluated.

The FCA rapidly simulates wire-based FFR values, albeit with certain simplifications. Contrarily, wire-based FFR values are derived from the complex physiological environment of the coronary vasculature. Considering the nature of the FCA, it does not perfectly match the FFR measured using a pressure wire. Based on the results of major validation studies, the accuracy of FCA falls between 0.85 and 0.95, indicating that the diagnostic discordance between FCA and FFR can occur in one or two out of 10 lesions [22, 25, 74]. However, these mismatches are frequently noted around the cutoff value of 0.80, where the efficacy of PCI on the reduction of serious clinical events such as death and MI might be modest if medical therapy is appropriately administrated [75]; when operators use FCA, careful consideration for the conduction of PCI is required, especially in borderline cases.

## Future perspectives of FCA

FCA is expected to improve its accuracy and reproducibility through automated procedures and elaborate computational algorithms. Several emerging technologies have unique features in their algorithms, generating functional information and successfully addressing the limitations of the FCA. Several novel software packages are equipped with functions that generate physiological parameters other than the FFR, such as the virtual NHPI and IMR [14, 15, 76]. These parameters potentially improve comprehensive physiological assessment in patients undergoing angiography, eliminating the need for additional diagnostic equipment such as the pressure wire. In future, the angiography system could evolve into a “one-stop shop,” seamlessly providing comprehensive anatomical and functional information, thereby reducing the dependence on additional diagnostic tools. Three notable technologies that are emerging in the field of FCA with their unique features are described below.

### µQFR

To address the limitations of conventional FCA (Table 1), a novel µQFR software (AngioPlus Core, Pulse Medical Imaging Technology Co., Ltd, Shanghai, China) has been developed [77]. The conventional FCA software requires multiple angiographic projections to reconstruct a 3D vessel model. Moreover, side branches cannot be integrated into the computational model, except for the FFRangio system. The µQFR system only requires a single angiographic projection to estimate the FFR value, considering the effects of outgoing flow toward the side branches and the step-down reference diameter in the interrogated vessel, according to Murray bifurcation fractal law. The delineation in the interrogated vessel and its side branches with ≥ 1.0 mm caliber is performed automatically by AI, allowing a fast computational time.

µQFR showed perfect feasibility (100%) and remarkable accuracy in detecting hemodynamically significant coronary stenosis when compared to wire-derived FFR as the reference (93%, 95% CI: 90.3–95.8%) in a retrospective validation study [77]. The average computational time only required 67 ± 22 s. In various specific clinical and anatomical subsets, the diagnostic accuracy of µQFR has also been substantiated such as severely calcified lesions and severe aortic stenosis [78–81]. The most notable advantage of the µQFR is its improved modeling for bifurcated vessels such as the left main coronary artery, indicating a stronger correlation of this technology with future clinical events. Wang et al. elucidated a significant association between residual ischemia, as determined by µQFR ≤ 0.80, and a higher incidence of cardiovascular death at 3 years after angiographically successful left-main bifurcation PCI [82].

### Angio-iFR

Angio-iFR (Philips, San Diego, CA, US) is a novel angiography-derived physiological assessment software that provides both iFR and FFR estimates according to a single angiographic projection [76]. This was developed as the first modality to simulate the NHPI of the instantaneous wave-free ratio (iFR). This development was based on the innovative concept that angiography-derived NHPI is more direct and accurate, as FCA is commonly calculated using angiography acquired under resting conditions [76]. The Angio-iFR algorithm requires a single projection and employs a common mathematical formula for pressure drop based on Poiseuille’s law. It utilizes a lumped parameter model for the boundary conditions.

The ReVEAL iFR study was a prospective validation study of the Angio-iFR, and the results were presented in EuroPCR 2023 [76, 83]. Among 485 target vessels, the analyzability was 97%, and the average computation time was 58 ± 22 s. The per-vessel sensitivity of Angio-iFR was 77%, which exceeded the prespecified performance goal of 75%, whereas the specificity was 49%, which did not reach the prespecified performance goal of 80%. The reason for this adverse outcome remains unclear. However, it is noteworthy that the core laboratory involved in this study also analyzed other FCA in the same dataset and found that the diagnostic accuracies were comparable to those of the Angio-iFR [84]. Further studies are warranted to clarify the true performance of the Angio-iFR software prior to its implementation in clinical practice.

### AutocathFFR

AutocathFFR (MedHub AI, Tel Aviv, Israel) was developed to allow a unique “hands-free” analysis approach. This technology eliminates manual procedures during analysis and improves the reproducibility of FCA. In addition, unlike other software which depend on CFD, AutocathFFR employs an “end-to-end” artificial neural network using a machine learning algorithm. This network was developed using numerous angiographic images from numerous training cases, allowing the software to accurately quantify the physiological significance of coronary artery stenosis using angiographic images only [17]. The development of this technology involved the training of a neural network with over 400 million individual cine frames. AutocathFFR can generate a virtual FFR of three (for left coronary arteries) or two (for right coronary artery) angiographic projections without the need for user operations, such as frame selection, lumen delineation, and frame counting, with a short procedure time (37 s). The first pilot study was conducted in 31 patients who underwent coronary angiography with wire-based FFR measurements. In this study, AutocathFFR showed excellent reproducibility with a correlation coefficient of 0.99, while its sensitivity, specificity, positive predictive value, and negative predictive value were 0.88, 0.93, 0.94, and 0.87, respectively. Thus, an accuracy level of 90% and an area under the curve of 0.91 were achieved [17].

### Perspectives on medical cost

The FCA potentially reduces medical costs by minimizing the chances of pressure wire use and mitigating unnecessary PCI. Medical insurance coverage for FCA use varies from country to country. This variation is mainly attributed to differences in health-care systems, insurance policies, and the regulatory status of medical technologies. In Japan, the current insurance policy covers FCA only along with diagnostic angiography, not angiography during PCI. Consequently, several important uses of FCA have not been covered. These included FCA assessment, followed by ad hoc PCI, immediately after PCI, and non-culprit lesion assessment. These applications crucially reflect the advantages of FCA, which potentially results in reduced medical costs, enhanced efficiency of coronary functional assessment, and improved patient safety. A future-focused policy approach in Japan and other countries could greatly benefit from expanding insurance coverage in alignment with global health-care trends and technological advancements in cardiology.

### Future role of coronary angiography in the context of coronary functionality

The scope of coronary angiography has expanded, highlighting not only on geometric assessment, but also on functional assessment with the advent of FCA. When the term “function” is referred to in the context of coronary artery, there are slightly different meanings: first, the role of conducting blood to the myocardium and second, active vasomotion which regulates coronary perfusion. The coronary angiography which is performed to assess abnormalities in coronary vasomotion using acetylcholine is also called “FCA” [85]. The state-of-the-art coronary angiography can evaluate two components of coronary functionality, which are the capacity of the epicardial artery to efficiently deliver blood to the myocardium, assessed through virtual FFR, and the vasomotion of the epicardial artery to modulate coronary flow, which is pivotal in diagnosing ischemic non-obstructive coronary artery disease, evaluated with intracoronary acetylcholine provocation testing. This comprehensive approach to coronary angiography is broadly referred to as FCA. However, these two roles of angiography in evaluating coronary function may be differentiated based on distinct names in future.

## Conclusions

FCA is a promising physiological assessment technology that eliminates the use of a pressure wire and, hence, increases the accessibility of quantified functional significance in the coronary arteries (Graphical Abstract). This next-generation functional vessel assessment entails seamless treatment decision-making before, during, and after revascularization. Improvements in technology have been iterated with unceasing efforts to increase its usability and diagnostic performance, including precision. Operators should be aware of the advantages and limitations associated with the current version of the FCA as a decision aid for revascularization. Thoughtful judgment is crucial when determining the course of revascularization by considering clinical perspectives such as the symptoms and backgrounds of the patients, particularly when the FCA falls within the intermediate zone.

## Data Availability

The data presented in this document is based on published data. Nevertheless, interested parties may request additional data from the corresponding author, and we will accommodate such requests to the best of our ability.
